# Supplementary material for: Dynamics of Transcription Factor Binding Site Evolution
Source: PLoS Genet. 2015 Nov 6;11(11):e1005639. doi: 10.1371/journal.pgen.1005639 (PMC4636380; doi:10.1371/journal.pgen.1005639)
Supplement: S1 Text — (PDF) [file pgen.1005639.s001.pdf]

Other fitness models for comparison & for interacting TFBSs

Power-law decaying fitness models for comparison: In order to understand the importance of the thermodynamically-motivated sigmoid shape for the binding probability, we compare our results to those obtained with power-law functions that decay with exponent γ (note that $\gamma = \infty$ corresponds to a step-like fitness landscape), formally defined as

$$\pi_{\text{pl}}(k) = \begin{cases} \pi_{\text{TD}}(k) & k \leq k_{\mathcal{S}} \\ \left(k_{\mathcal{S}}/k\right)^{\gamma} \pi_{\text{TD}}(k_{\mathcal{S}}) & k > k_{\mathcal{S}} \end{cases}. \tag{1}$$

S3 Fig shows that the power-law exponent is a major determinant of the gain rates, suggesting that a biophysically realistic fitness landscape is crucial for the quantitative understanding of TFBS evolution.

Fitness models of interacting TFBSs in larger regulatory sequence: In addition to physical cooperativity between nearby TFs on promoter/enhancers (see Methods, Fig 5 and S8 Fig), here we also consider two other models. The first additional model assumes that the binding occupancy of the strongest binding site in the regulatory sequence is the proxy for the gene expression level and the fitness, i.e.

$$f(\boldsymbol{\sigma}) = s\,\text{MAX}\{\pi^{(\text{i})}(\boldsymbol{\sigma})\}. \tag{2}$$

Note that different TFBSs interact with each other to compete for the strongest binding within a promoter or an enhancer.

The second additional model addresses synergistic interaction between the two strongest-binding TFBS, located anywhere in the regulatory sequence. This example is a simplified version of a biophysical model where TFs, binding anywhere in a regulatory region, compete for the occupancy of that region with a nucleosome (for a more elaborative modeling framework, see Mirny (2010) [1]). We call this type of interaction between two TFs “non-physical” because TFs don’t interact directly; their interaction is effectively mediated by some other biophysical process. The probability of the joint occupancy of the two TFs at promoter or enhancer can be used as the proxy for gene expression level and the fitness, i.e.

$$f(\boldsymbol{\sigma}) = s\,\frac{e^{-\beta(\epsilon(k_1+k_2)-2\mu)}}{1+e^{-\beta(\epsilon k_1-\mu)}+e^{-\beta(\epsilon k_2-\mu)}+e^{-\beta(\epsilon(k_1+k_2)-2\mu)}}, \tag{3}$$

where k_1 and k_2 correspond to the genotypes of two TFBSs with the smallest mismatches in the regulatory sequence.

Do these models yield different result for the emergence of strong binding sites from random sequences at early evolutionary times ($\sim$ speciation time scales), in comparison to our main model, where the sum of binding occupancies is used as a proxy for gene expression level [Eq(7) in the main text]? For typical biophysical parameters (binding lenght: $n = 7$ bp, binding specificity: $\epsilon = 2\ k_BT$ and chemical potential: $\mu = 4\ k_BT$), we show in S9 Fig that these modified models do not differ extensively from results of our main model.

References

1. Mirny LA. Nucleosome-mediated cooperativity between transcription factors. Proceedings of the National Academy of Sciences. 2010 Dec;107(52):22534–22539.
