# Supplementary figures and images for: Dynamics of Transcription Factor Binding Site Evolution
Source: PLoS Genet. 2015 Nov 6;11(11):e1005639. doi: 10.1371/journal.pgen.1005639 (PMC4636380; doi:10.1371/journal.pgen.1005639)

a

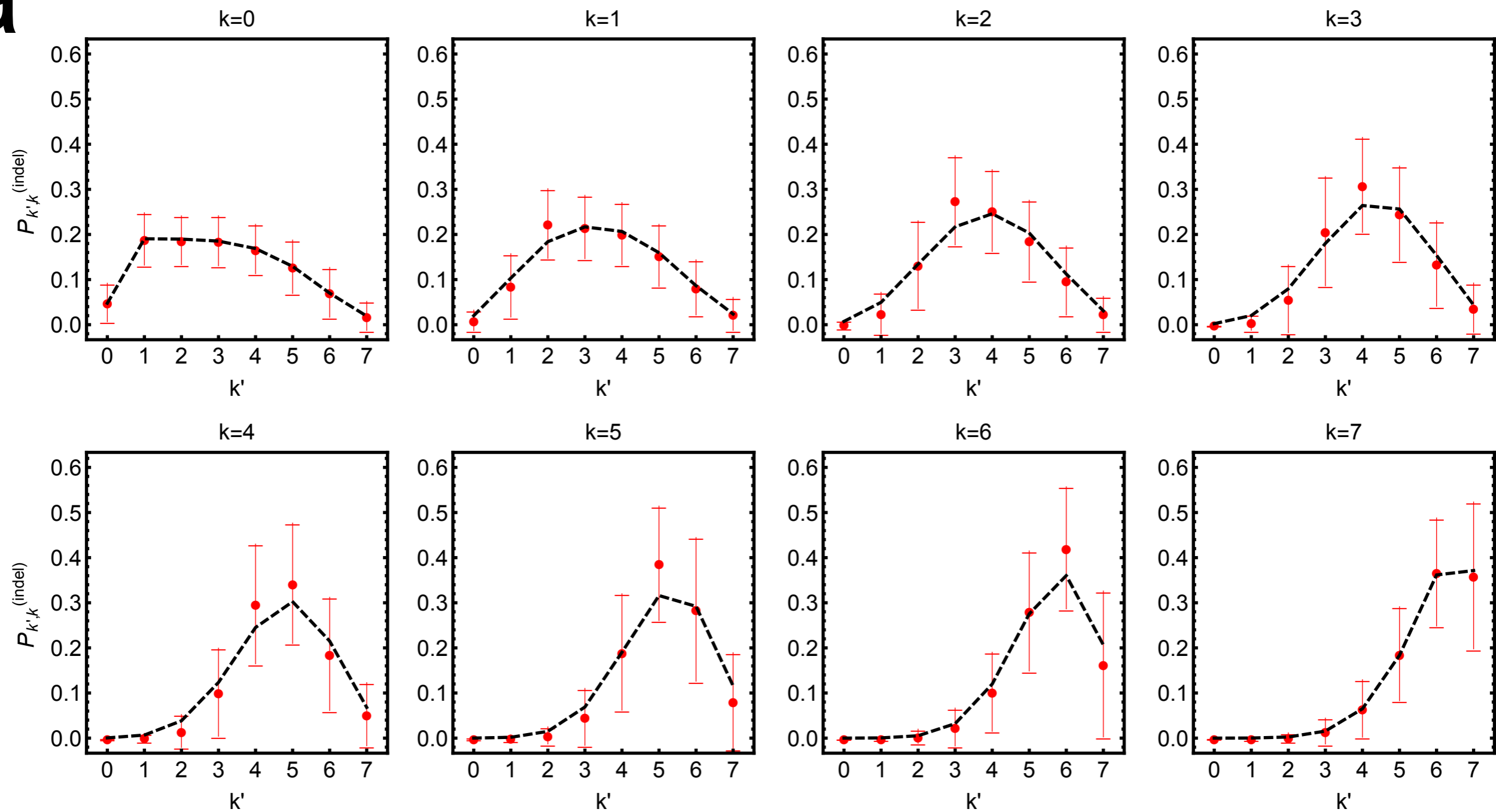

b

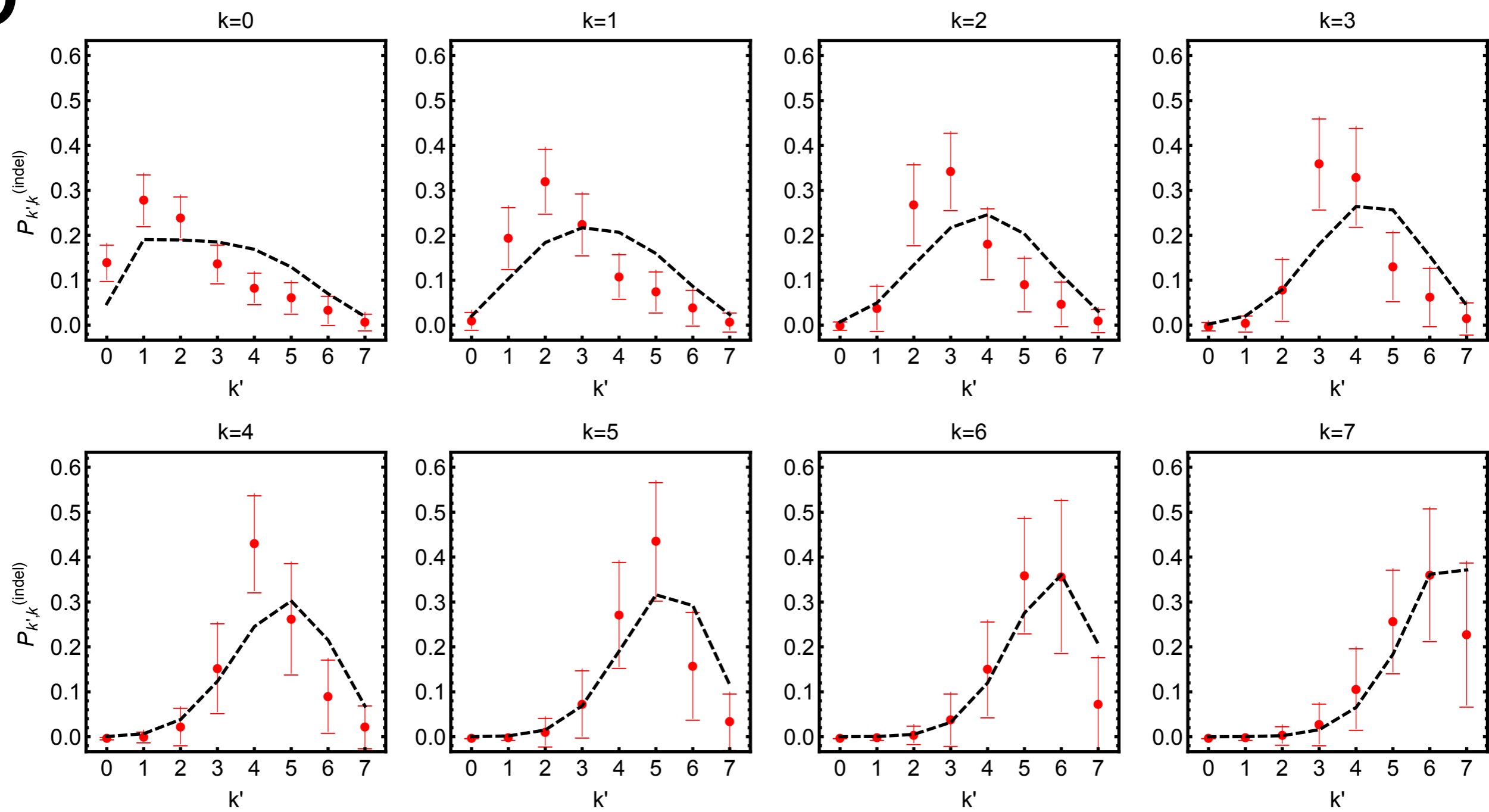

Supplement: S1 Fig — a) Probability that a binding site with k mismatches mutates to k′ mismatches, for a single binding site of length n = 7 bp, according to our indel mutation model in a fixed genomic window (see the Methods section). Dashed curve = analytical prediction according to Eq (13). Red points = mean ±1 std of 103 replicate realizations of the frequency distribution (for each replicate, 1 consensus sequence is created and 104 mutations are simulated for each k). b) The same analysis as in a), but allowing for a flexible genomic window for alignment after insertion mutations. We pick the minimal mismatch case to asses the quality of our approximation. As expected, this creates a bias towards smaller mismatch classes, but suggests that our approximation is still reasonable. (PDF) [file pgen.1005639.s002.pdf]

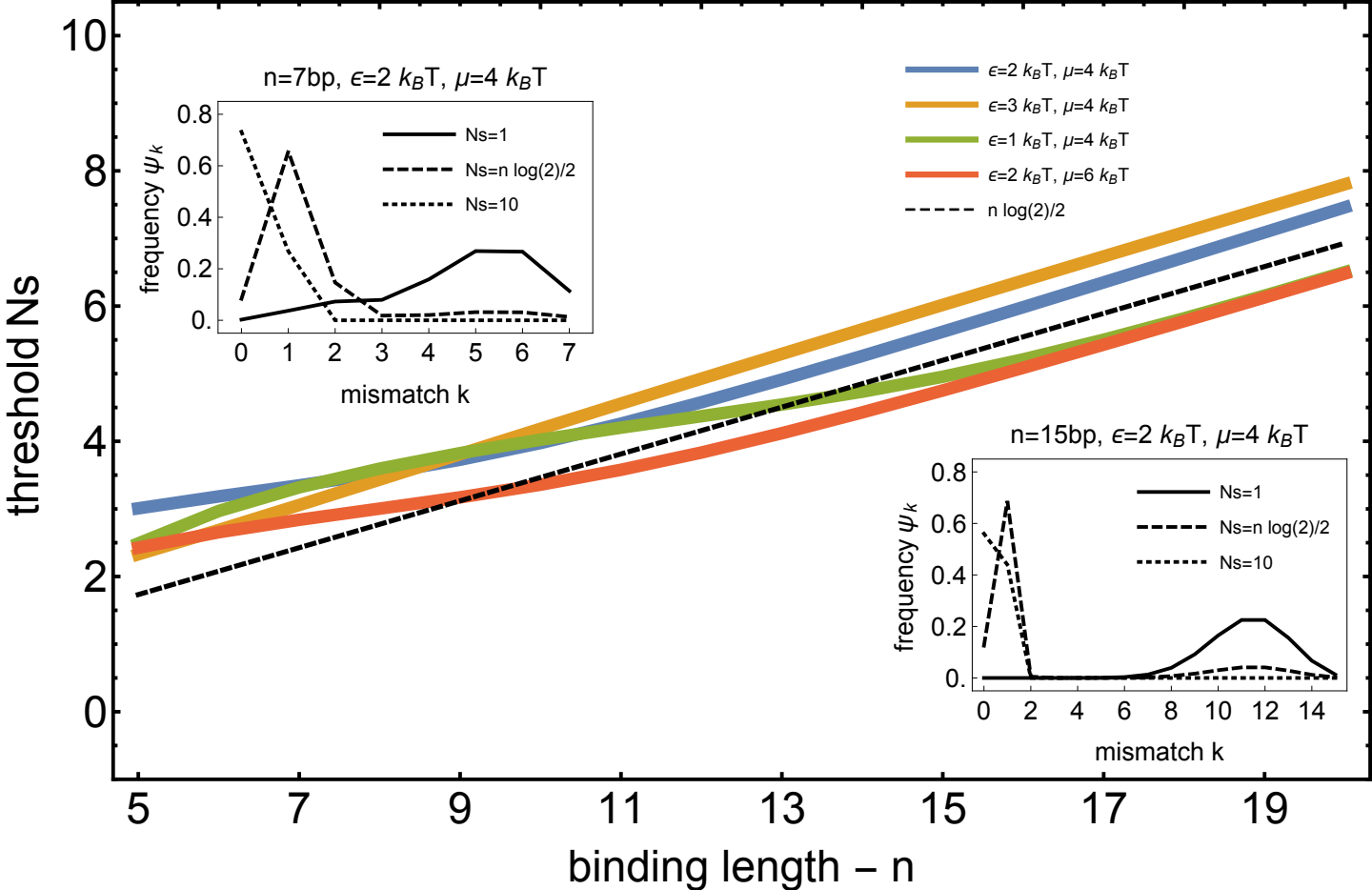

Supplement: S2 Fig — The value of Ns at which 5% of the probability weight in the stationary distribution is in non-strong mismatch classes, i.e. k > k 𝓢. For selection stronger than this threshold, the stationary distribution is concentrated at low k (high fitness) classes and is practically unimodal. Different colors correspond to different biophysical parameters (see legend), analytical prediction n log(2)/2 is in black (see the Methods section and Eq (20)). Insets show examples of stationary distributions for different Ns values for short and long binding sites. (PDF) [file pgen.1005639.s003.pdf]

**a**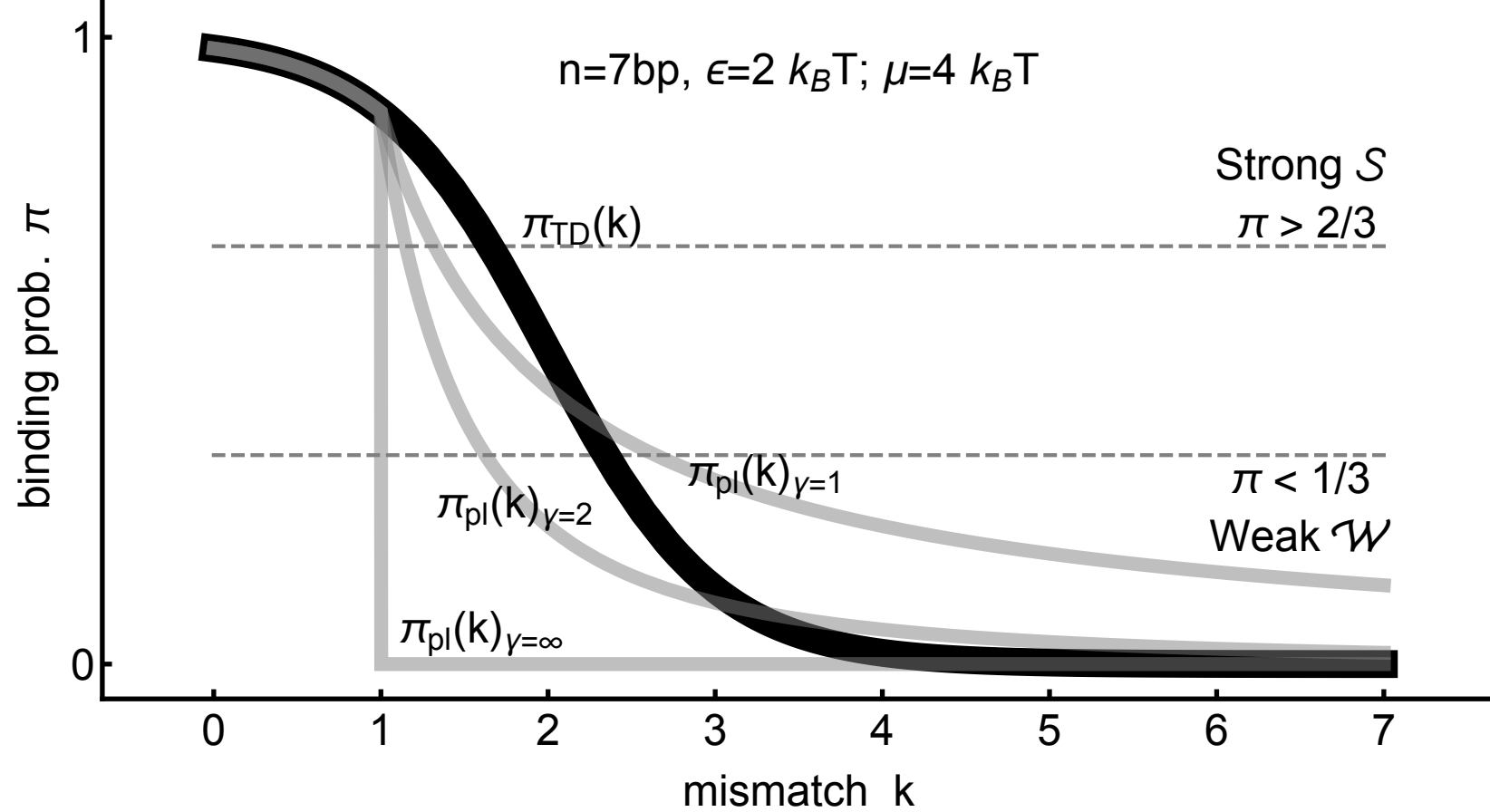**b**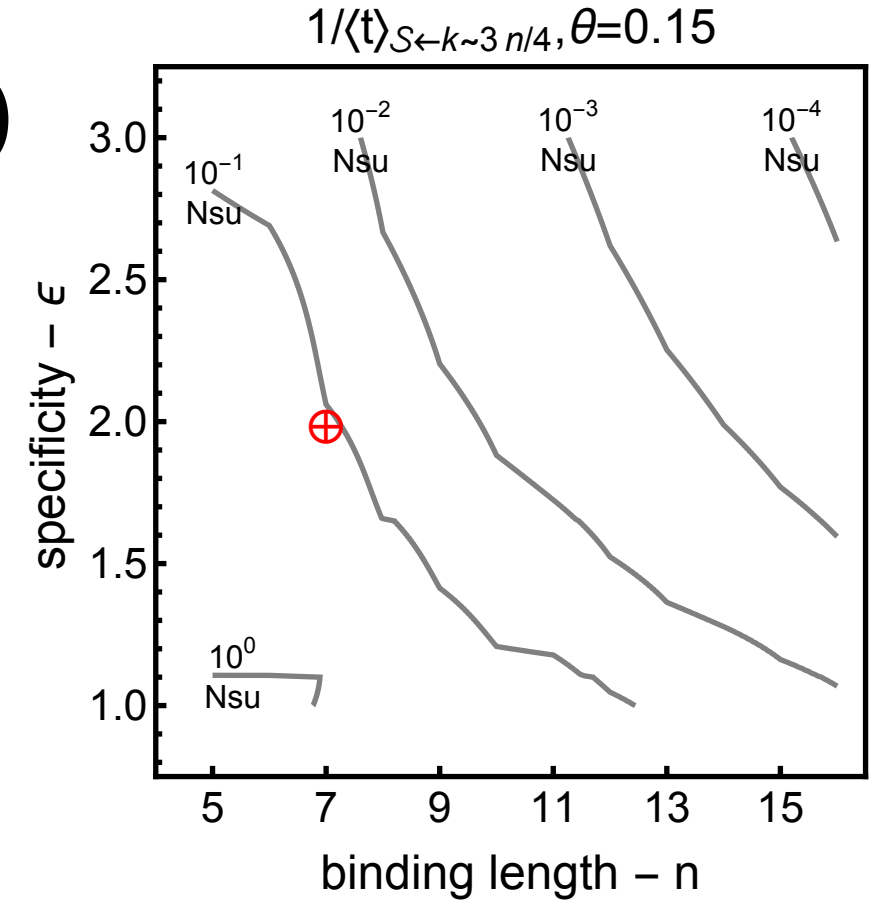**c**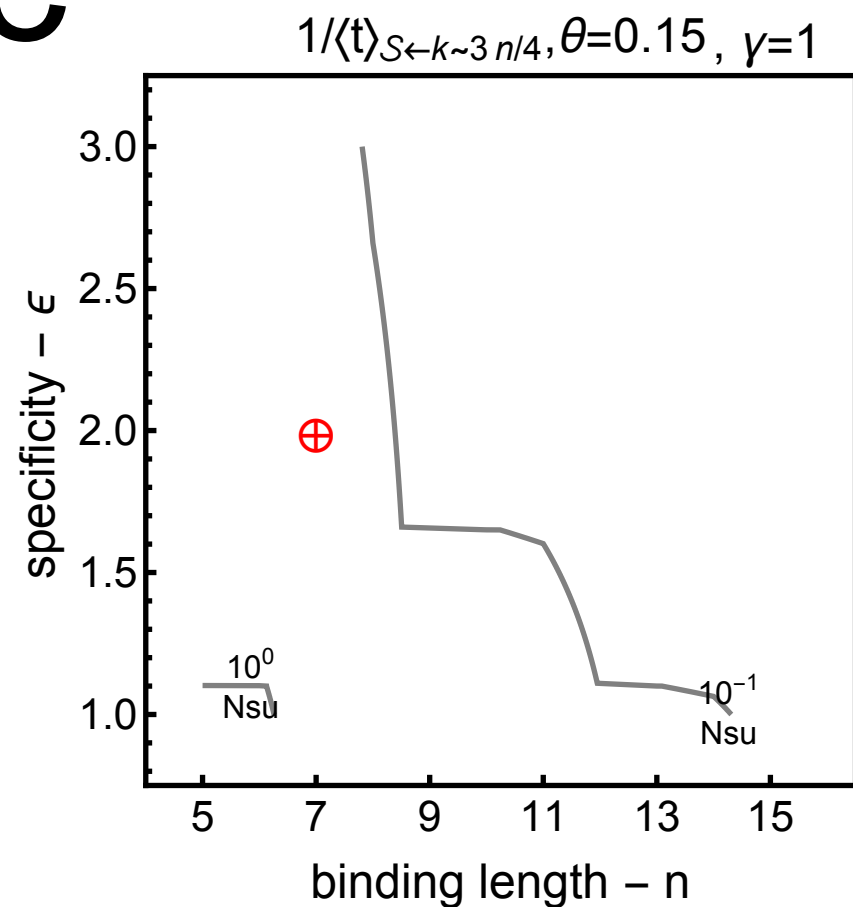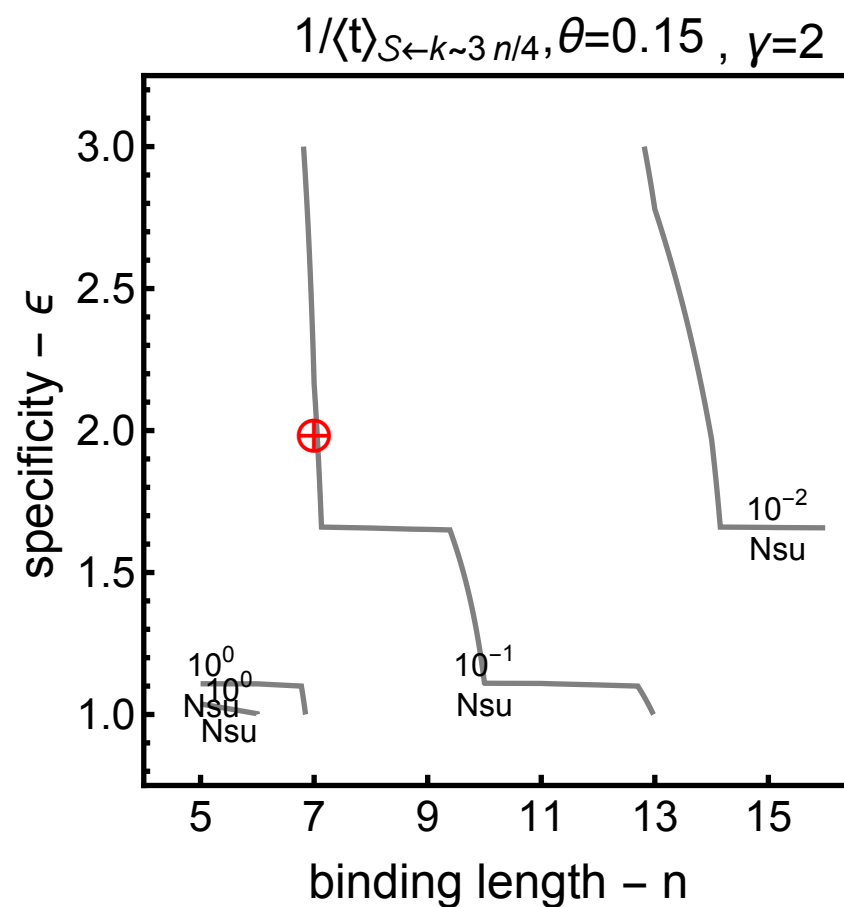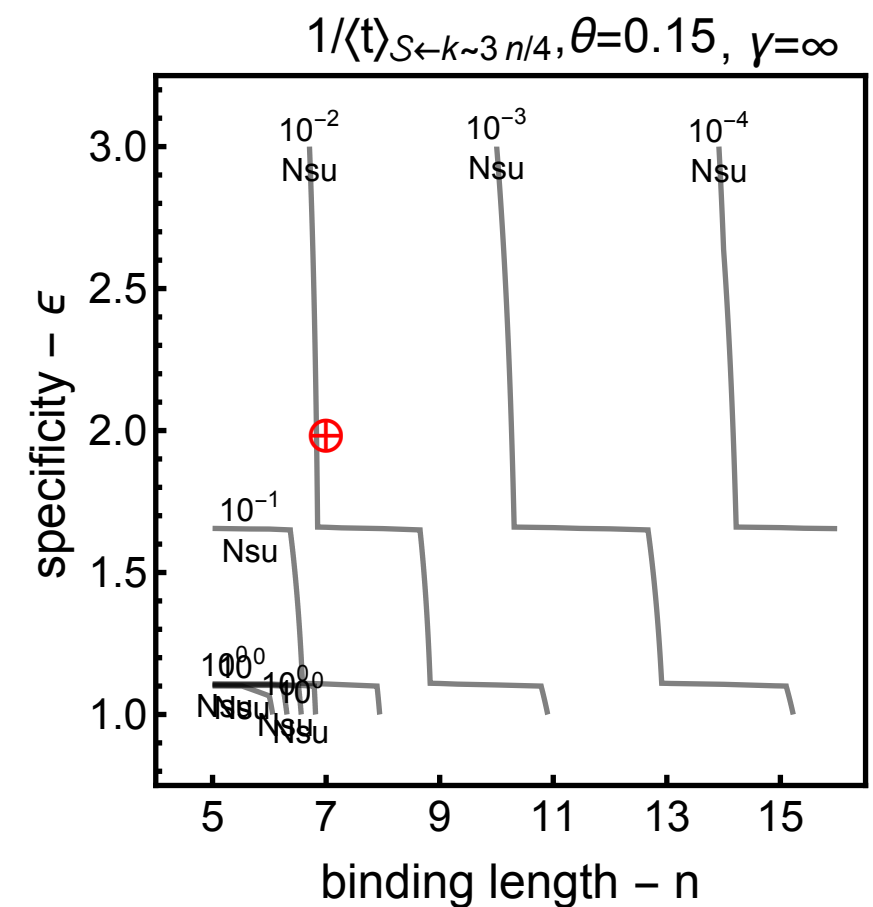

Supplement: S3 Fig — a) The thermodynamic fitness landscape has been modified to have a power-law decaying tail of exponent γ for k > k 𝓢, as in Eq (1) in S1 Text. We tested γ = 1, 2 and ∞ corresponding to smooth, intermediate and step-like decay. Plot conventions are the same as in Fig 2C. b) Isolated TFBS gain rate from the most redundant mismatch class for the thermodynamic model, replotted from Fig 2C for reference. c) Plots analogous to b) using modified fitness landscapes defined by the power-law exponent γ. Gain rates are higher for small γ = 1 and lower for the step landscape (γ = ∞), relative to the reference. (PDF) [file pgen.1005639.s004.pdf]

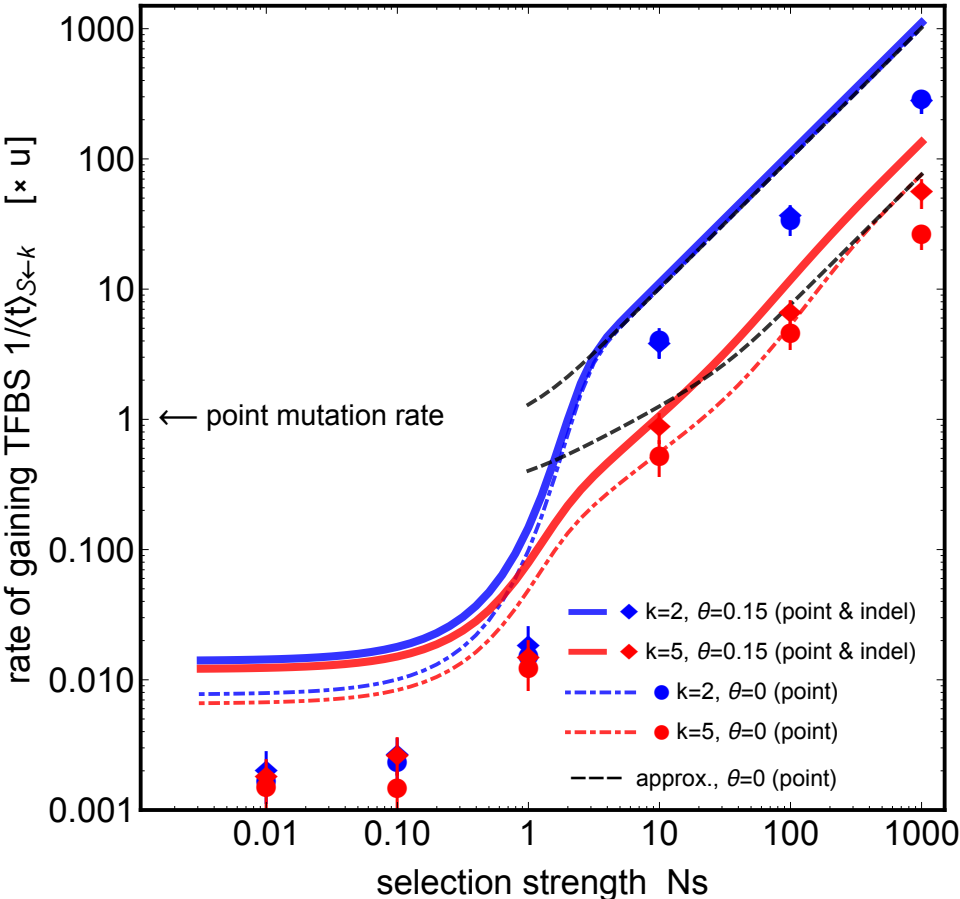

Supplement: S4 Fig — Wright-Fisher simulation results (point markers, error bars = 2 standard errors of the mean) at 4Nu = 0.1, in comparison to the fixed state model (continuous curves). Plot conventions are the same as in Fig 2. Biophysical parameters used: n = 7, ϵ = 2 k B T, μ = 4 k B T. Polymorphisms generally decrease TFBS gain rates. (PDF) [file pgen.1005639.s005.pdf]

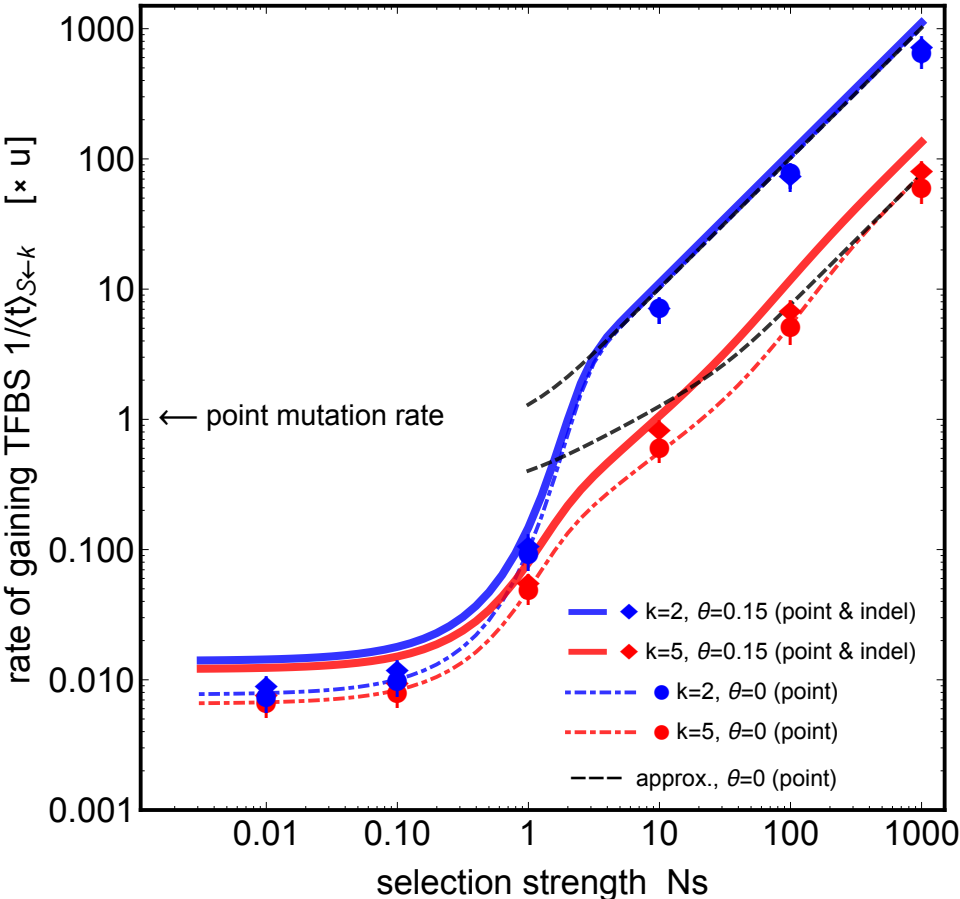

Supplement: S5 Fig — Fig 2, but using energy matrices whose nonzero entries are gaussian random variables ɛ i, such that ⟨ɛ i⟩ = ϵ = 2k B T and σ ɛ = 0.5k B T; n = 7, μ = 4k B T. The analytical results under the equal mismatch assumption are shown in continuous lines. (PDF) [file pgen.1005639.s006.pdf]

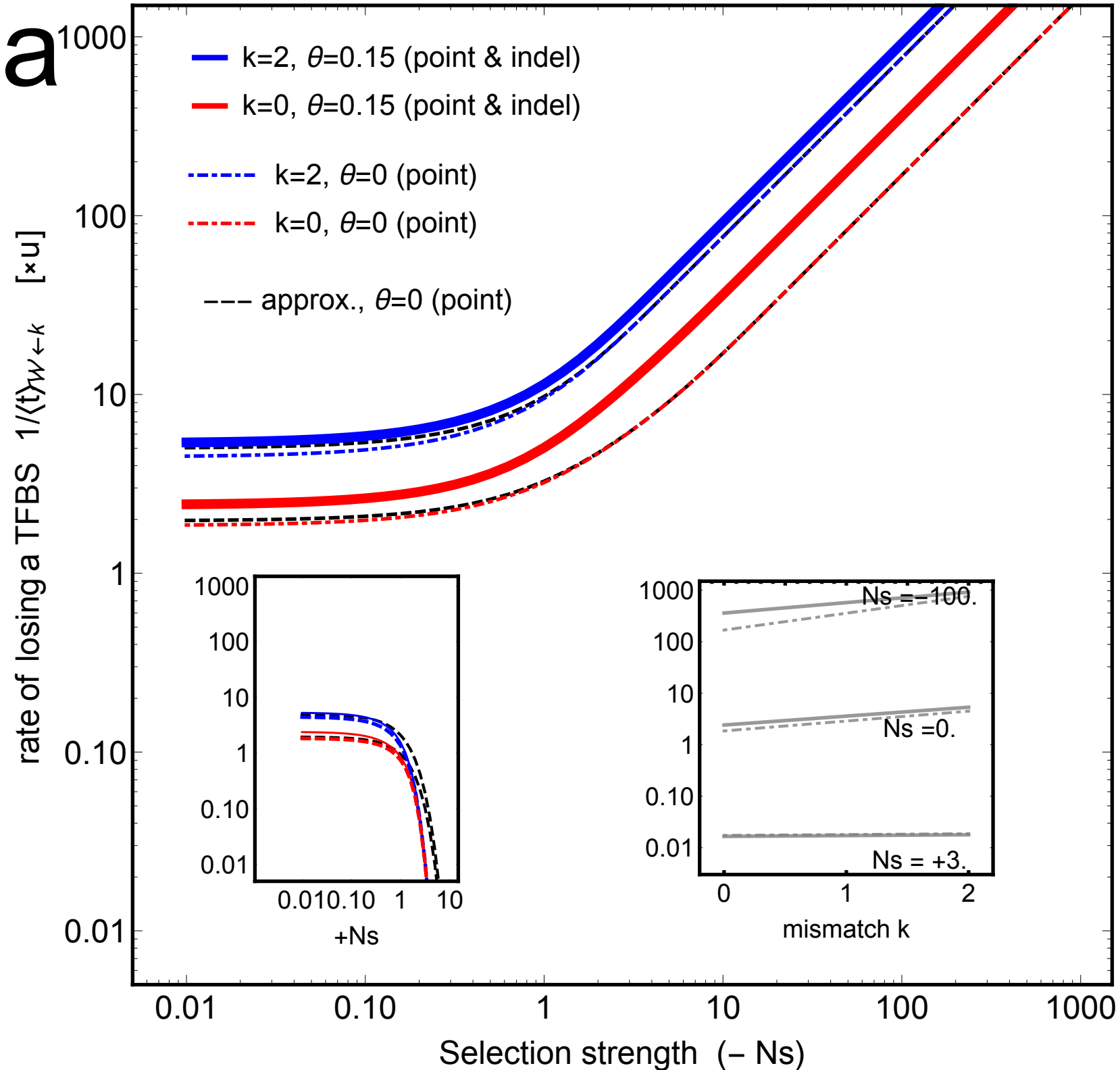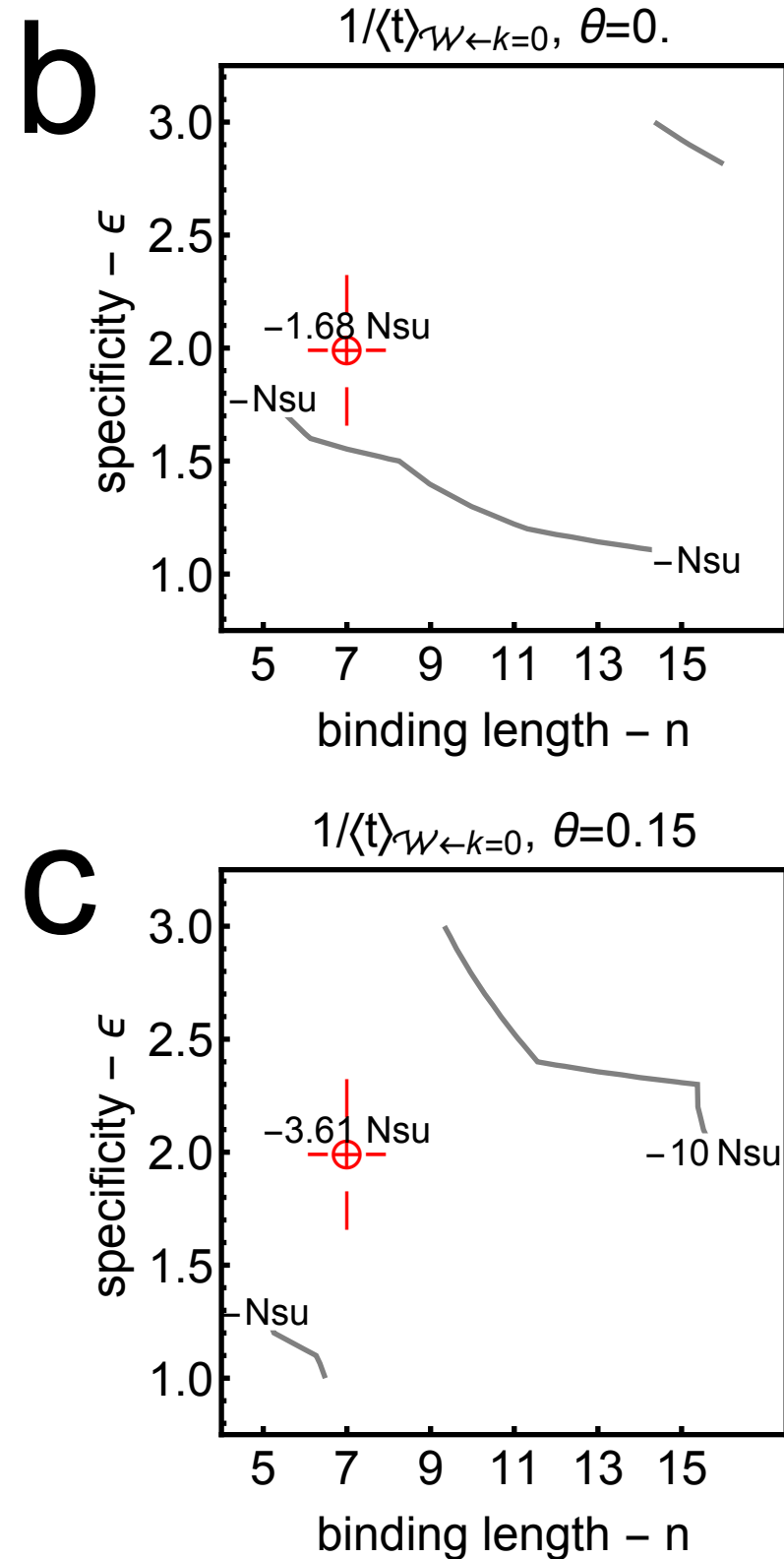

Supplement: S6 Fig — The dependence of the loss rate, 1/⟨t⟩𝓦 ← k shown in units of point mutation rate, from sequences in different initial mismatch classes k (blue: k = 2, red: k = 0), as a function of negative selection strength. Results with point mutations only (θ = 0) are shown by dashed line; with admixture of indel mutations (θ = 0.15) by a solid line. For strong selection, ∣Ns∣ ≫ 1, the rates scale with 2∣Ns∣nu, which is captured well by the “shortest path” approximation (black dashed lines in the main figure) of Eq (24). The biophysical parameters are: site length n = 7 bp; binding specificity ϵ = 2 k B T; chemical potential μ = 4 k B T. Left inset: Ns-scaling with positive selection. Right inset: gain rates as a function of the initial mismatch class k for different Ns. b, c) Loss rates from the consensus sequence (k = 0) under strong negative selection, without (b) and with (c) indel mutations supplementing point mutations. Red crosshairs denote the cases depicted in panel a). Contour lines show constant loss rates in units of Ns u as a function of biophysical parameters n and ϵ. (PDF) [file pgen.1005639.s007.pdf]

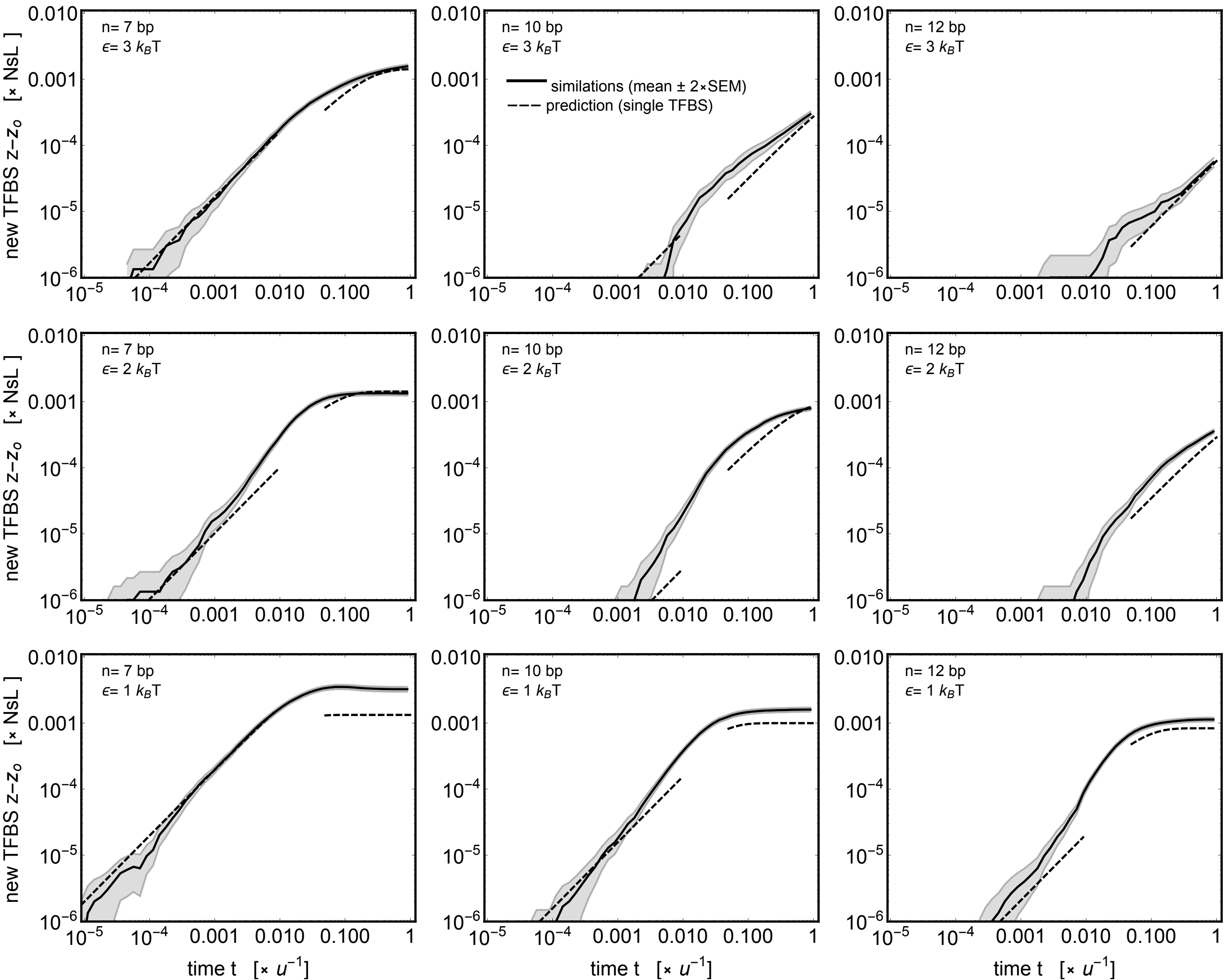

Supplement: S7 Fig — Example simulations (black solid line) and analytic predictions based on single TFBS gain/loss rates (black dashed line), for different binding length n and specificity ϵ. Details are identical to Fig 4. (PDF) [file pgen.1005639.s008.pdf]

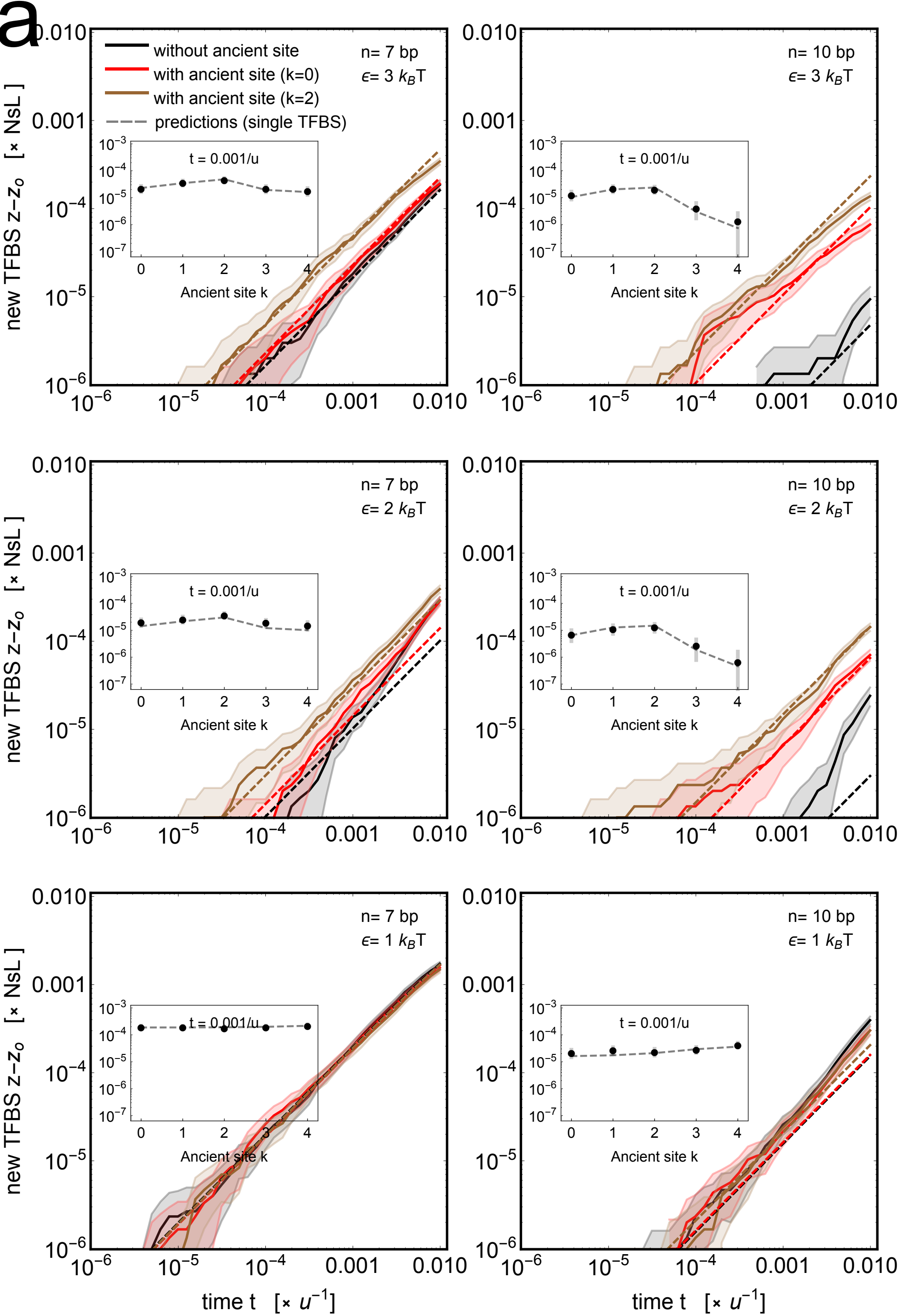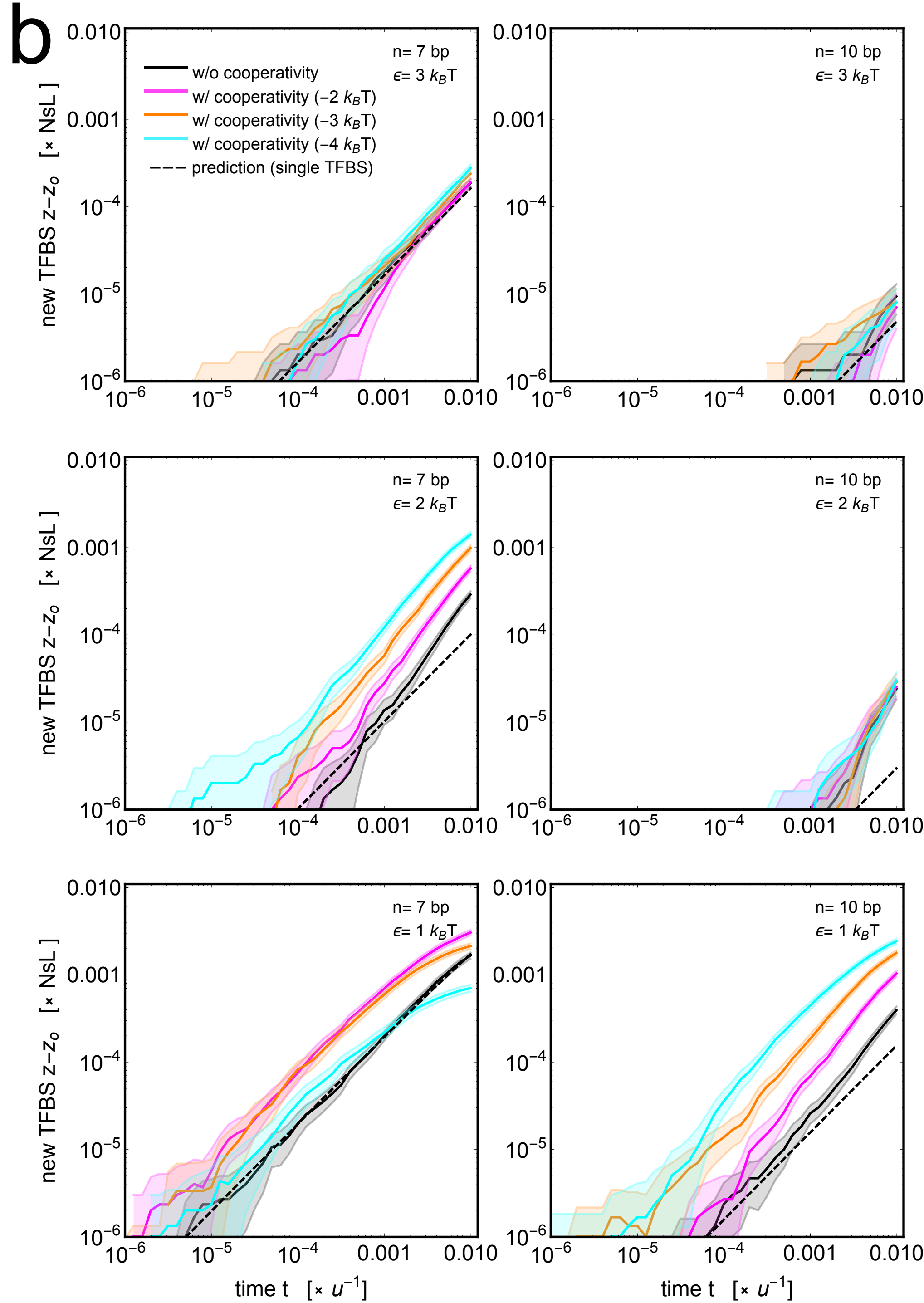

Supplement: S8 Fig — Simulations of TFBS evolution in longer sequences (colored lines) and analytic predictions based on single TFBS gain and loss rates (dashed black lines), analogous to Fig 5. Different panels show different choices of TFBS binding length n and specificity ϵ. Ancient sites specifically facilitate the emergence of longer sites of high specificity, whereas cooperativity specifically facilitates the emergence of shorter sites of intermediate or low specificity. (PDF) [file pgen.1005639.s009.pdf]

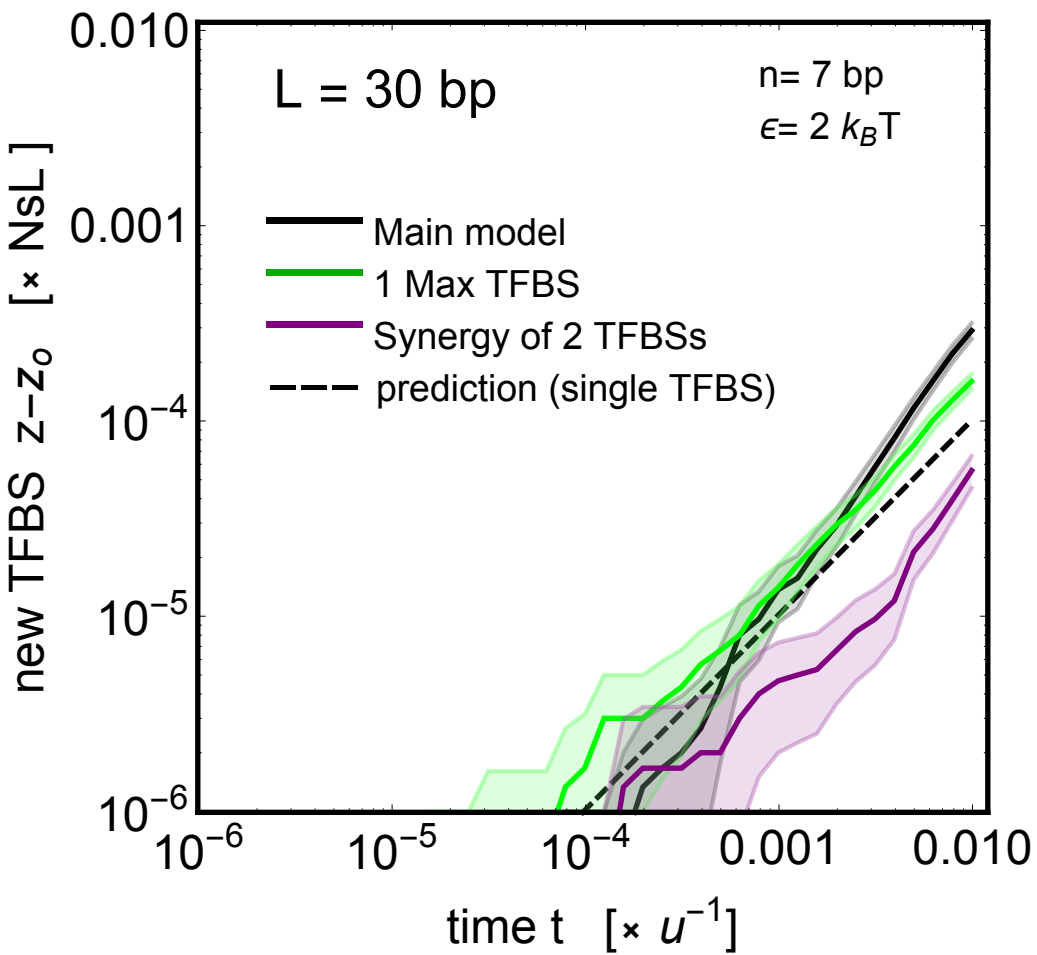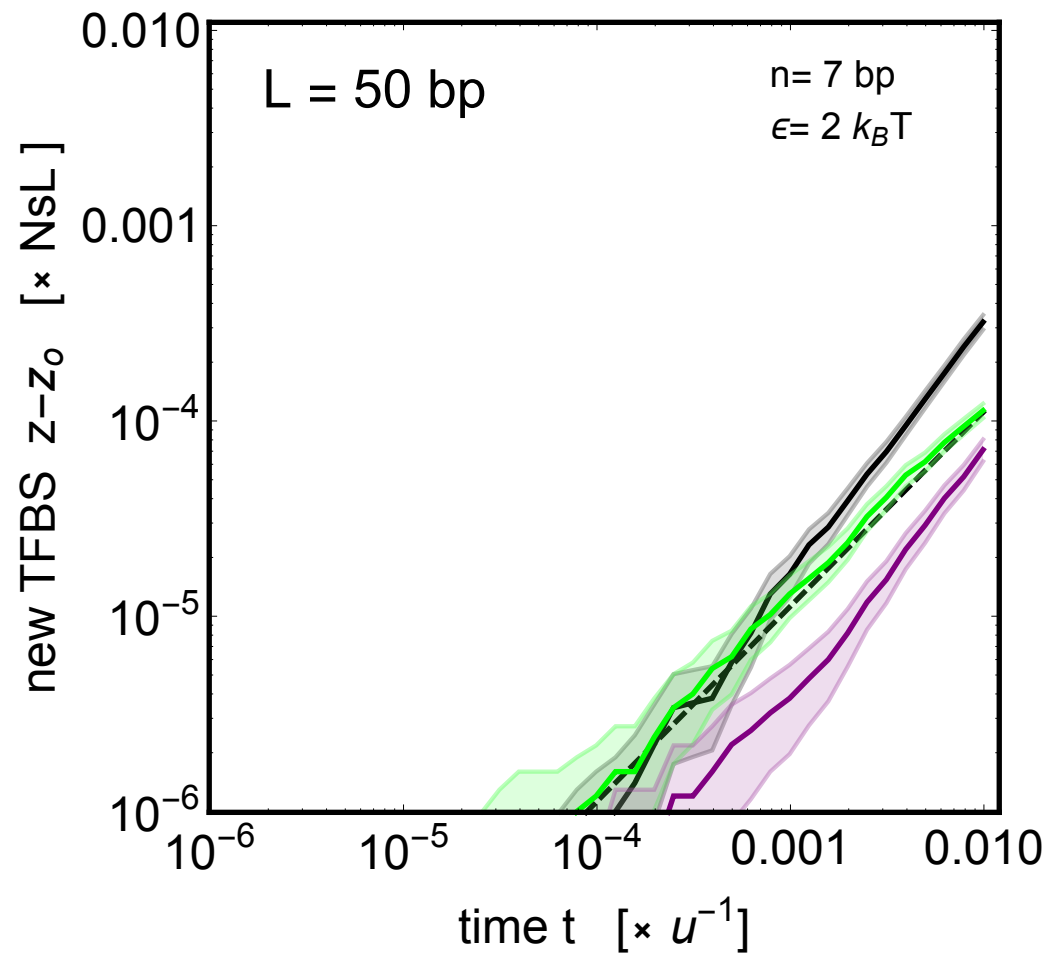

Supplement: S9 Fig — The expected number of newly evolved TFBS for binding site length n = 7 bp, specificity ϵ = 2 k B T, and chemical potential μ = 4 k B T are shown for different fitness models. The solid black curve is the non-interacting model used in the main text (dashed curve: theoretical prediction). The green curve stands for the model of Eq (2) in S1 Text, where only the strongest binding site in the regulatory sequence determines gene expression. The purple curve stands for the model of Eq (3) in S1 Text, where two strongest TFBS synergistically determine the gene expression level. Shading denotes ±2 SEM. The simulations use regulatory sequences of length L = 30 bp (left) and L = 50 bp (right). (PDF) [file pgen.1005639.s010.pdf]

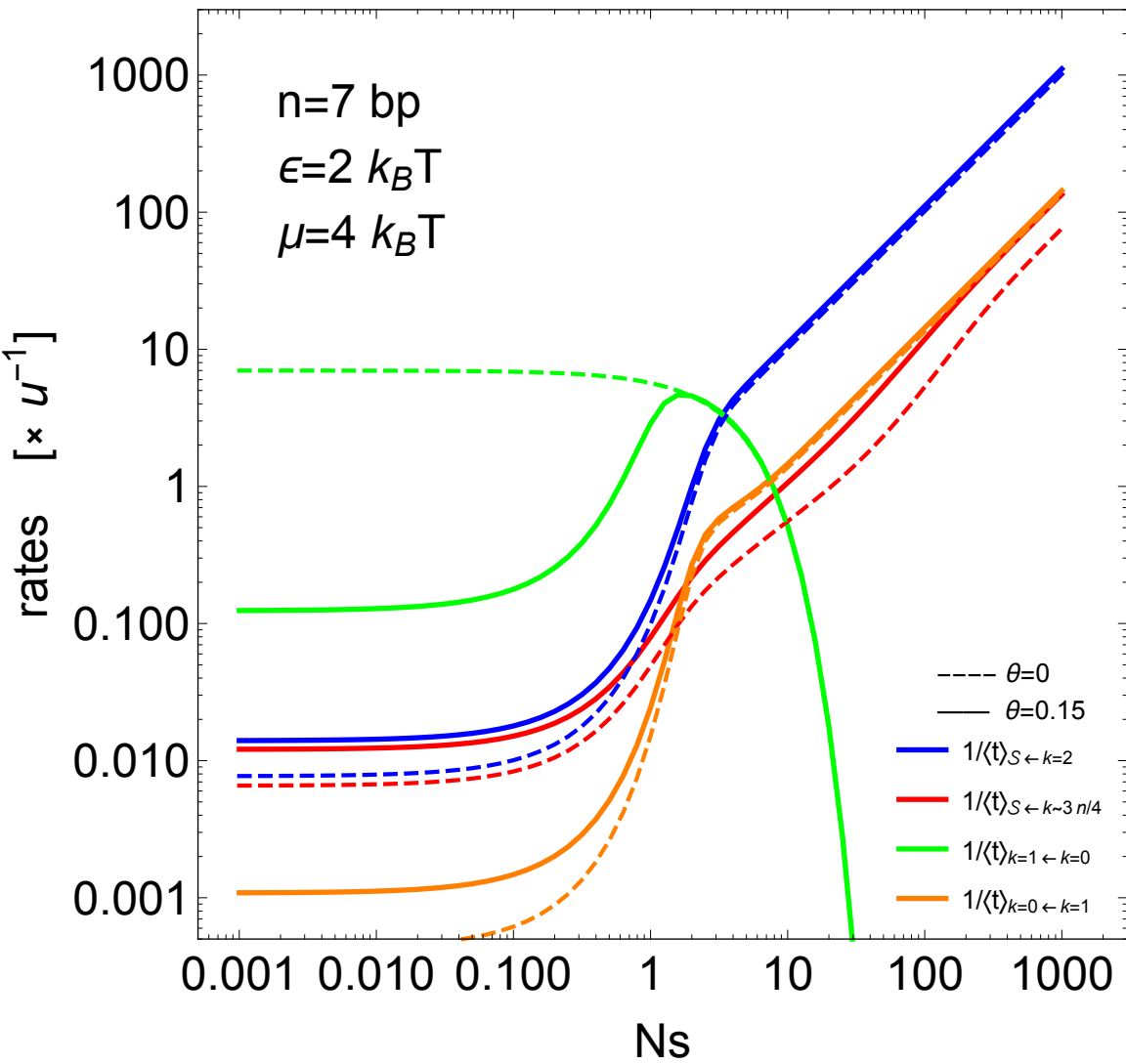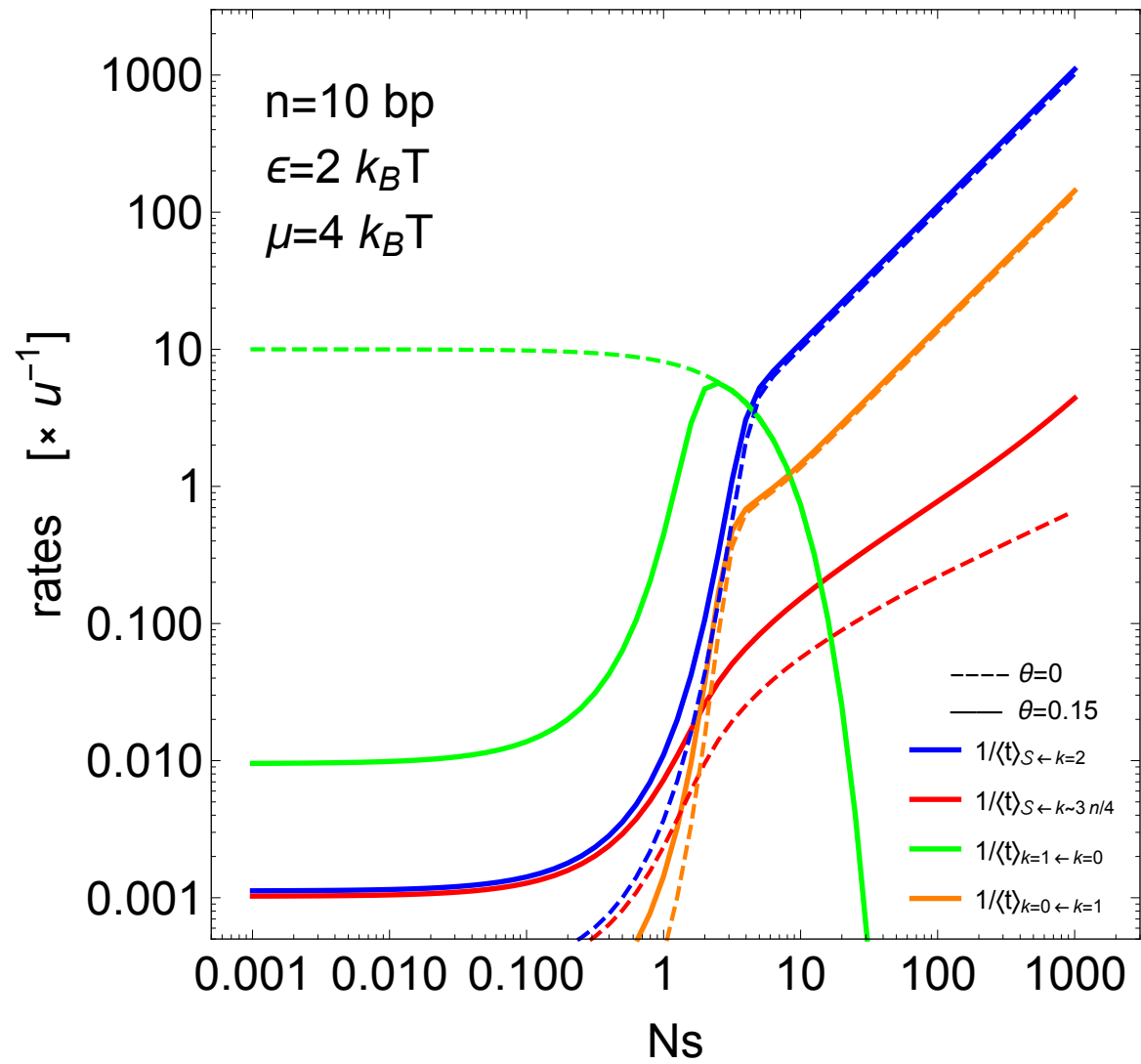

Supplement: S10 Fig — Average first hitting times to particular mismatch k j state can be calculated with a minor modification to Eq (21) by replacing 𝓢 with k j. The figures compare the rates of evolution of TFBS within the functional sites (i.e. 1/⟨t⟩k = 0 ← k = 1 and 1/⟨t⟩k = 1 ← k = 0). Plot conventions are the same as in Fig 2A. Biophysical parameters used: n = 7 bp (left), n = 10 bp (right) ϵ = 2 k B T, μ = 4 k B T. It shows that for weak selection, the rates to evolve from k = 0 to k = 1 can be relatively faster. Also, although adaptation from random sites slows down with increasing n, we see that the adaptation rate to evolve from k = 1 to k = 0 can stay high. (PDF) [file pgen.1005639.s011.pdf]
